# Supplementary material for: Polyketide synthase gene clusters in the ericoid mycorrhizal fungus Oidiodendron maius: genome-wide prediction, expression profiling, and functional characterization of knockout mutants
Source: Front Plant Sci. 2026 Sep 16;17:1924430. doi: 10.3389/fpls.2026.1924430 (PMC13623903; doi:10.3389/fpls.2026.1924430)
Supplement: Supplementary file 1 [file SupplementaryFile1.zip › Supplementary Materials/Supplementary Folder 1.zip!/Oidma1_AssemblyScaffolds_Repeatmasked/knownclusterblast/cluster42/jgi.p_Oidma1_183700_mibig_hits.html]

| MIBiG Protein | Description | MIBiG Cluster | MiBiG Product | % ID | % Coverage | BLAST Score | E-value |
| --- | --- | --- | --- | --- | --- | --- | --- |
| BAE56591.1 |  | BGC0001123 | Aspirochlorine\_biosynthetic\_gene\_cluster | 56 | 92.3954372624 | 578 | 8.9e-165 |
| AAC49200.1 | putative\_p450\_monooxygenase | BGC0000152 | Sterigmatocystin\_biosynthetic\_gene\_cluster | 39 | 92.0152091255 | 346 | 7.5e-95 |
| AAS90059.1 | VerB | BGC0000009 | Aflatoxin\_biosynthetic\_gene\_cluster | 39 | 94.1064638783 | 344 | 2.8e-94 |
| OAQ83755.1 | isotrichodermin\_C-15\_hydroxylase | BGC0001358 | Leucinostatins\_biosynthetic\_gene\_cluster | 38 | 91.6349809886 | 339 | 5.3e-93 |
| AAK53587.1 | isotrichodermin\_C-15\_hydroxylase | BGC0000931 | Trichothecene\_biosynthetic\_gene\_cluster | 39 | 93.1558935361 | 339 | 7e-93 |
| AAK33080.1 | putative\_trichothecene\_C-15\_hydroxylase | BGC0001277 | Nivalenol\_/\_deoxynivalenol\_/\_3-acetyldeoxynivalenol\_/ | 39 | 93.1558935361 | 337 | 2.7e-92 |
| AAK53580.1 | isotrichodermin\_C-15\_hydroxylase | BGC0000930 | Trichothecene\_biosynthetic\_gene\_cluster | 39 | 93.1558935361 | 337 | 2.7e-92 |
| BAK26559.1 | cytochrome\_P450 | BGC0000977 | Cyclopiazonic\_acid\_biosynthetic\_gene\_cluster | 36 | 94.1064638783 | 337 | 2.7e-92 |
| BAE71326.1 | desaturase | BGC0000004 | Aflatoxin\_biosynthetic\_gene\_cluster | 38 | 94.1064638783 | 336 | 4.5e-92 |
| AAS90011.1 | VerB | BGC0000007 | Aflatoxin\_biosynthetic\_gene\_cluster | 39 | 94.1064638783 | 336 | 4.5e-92 |
| AAS90033.1 | VerB | BGC0000008 | Aflatoxin\_biosynthetic\_gene\_cluster | 39 | 94.1064638783 | 336 | 5.9e-92 |
| AAS90102.1 | VerB | BGC0000006 | Aflatoxin\_biosynthetic\_gene\_cluster | 38 | 94.1064638783 | 336 | 7.7e-92 |
| AAS90079.1 | VerB | BGC0000010 | Aflatoxin\_biosynthetic\_gene\_cluster | 38 | 94.1064638783 | 336 | 7.7e-92 |
| AAK33070.1 | trichothecene\_C-15\_hydroxylase | BGC0001278 | Nivalenol\_/\_deoxynivalenol\_/\_3-acetyldeoxynivalenol\_/ | 39 | 92.2053231939 | 334 | 2.2e-91 |
| XP\_002384857.1 | cytochrome\_P450,\_putative | BGC0001234\_c2 | 4,4'-piperazine-2,5-diyldimethyl-bis-phenol\_biosynthetic\_gene | 38 | 93.1558935361 | 333 | 3.8e-91 |
| AAM18769.1 | AflL | BGC0000011 | Aflatoxin/sterigmatocystin\_biosynthetic\_gene\_cluster | 38 | 91.6349809886 | 329 | 5.5e-90 |
| ACH72905.1 | AflG | BGC0000011 | Aflatoxin/sterigmatocystin\_biosynthetic\_gene\_cluster | 40 | 90.8745247148 | 328 | 1.6e-89 |
| BAD83682.1 | cytochrome\_P-450 | BGC0000012 | Alternapyrone\_biosynthetic\_gene\_cluster | 38 | 91.0646387833 | 326 | 6.1e-89 |
| ACB12551.1 | Fum2 | BGC0000063 | Fumonisin\_biosynthetic\_gene\_cluster | 36 | 93.1558935361 | 322 | 8.8e-88 |
| ACD39760.1 | cytochrome\_P450 | BGC0000077 | Hypothemycin\_biosynthetic\_gene\_cluster | 37 | 93.536121673 | 320 | 3.4e-87 |
| ACD39751.1 | cytochrome\_P450 | BGC0000076 | Hypothemycin\_biosynthetic\_gene\_cluster | 37 | 93.536121673 | 319 | 7.5e-87 |
| AAC49196.1 | putative\_p450\_monooxygenase | BGC0000152 | Sterigmatocystin\_biosynthetic\_gene\_cluster | 40 | 86.8821292776 | 315 | 1.1e-85 |
| AAN74815.2 | Fum2p | BGC0000062 | Fumonisin\_biosynthetic\_gene\_cluster | 36 | 89.7338403042 | 313 | 4.1e-85 |
| AAS90078.1 | AvnA | BGC0000010 | Aflatoxin\_biosynthetic\_gene\_cluster | 39 | 90.8745247148 | 311 | 1.6e-84 |
| AAS90101.1 | AvnA | BGC0000006 | Aflatoxin\_biosynthetic\_gene\_cluster | 39 | 90.8745247148 | 311 | 2e-84 |
| AAS90010.1 | AvnA | BGC0000007 | Aflatoxin\_biosynthetic\_gene\_cluster | 39 | 90.8745247148 | 311 | 2.7e-84 |
| AAS90032.1 | AvnA | BGC0000008 | Aflatoxin\_biosynthetic\_gene\_cluster | 39 | 90.8745247148 | 310 | 3.5e-84 |
| EFY94501.1 | benzoate\_4-monooxygenase\_cytochrome\_P450 | BGC0000337 | Destruxins\_biosynthetic\_gene\_cluster | 37 | 93.7262357414 | 310 | 4.5e-84 |
| BAE71325.1 | cytochrome\_P450\_monooxygenase | BGC0000004 | Aflatoxin\_biosynthetic\_gene\_cluster | 39 | 90.8745247148 | 309 | 1e-83 |
| AAS90058.1 | AvnA | BGC0000009 | Aflatoxin\_biosynthetic\_gene\_cluster | 39 | 90.8745247148 | 309 | 1e-83 |
| BAD29972.1 | P450\_monooxygenase\_1 | BGC0000676 | Aphidicolin\_biosynthetic\_gene\_cluster | 36 | 93.7262357414 | 303 | 4.2e-82 |
| BAQ25465.1 | cytochrome\_P450 | BGC0001264 | Betaenone\_A\_/\_betaenone\_B\_/\_betaenone\_C\_biosynthetic\_gene\_cluster | 36 | 94.2965779468 | 302 | 7.2e-82 |
| ADI24937.1 | VrtK | BGC0000168 | Viridicatumtoxin\_biosynthetic\_gene\_cluster | 34 | 89.1634980989 | 296 | 5.2e-80 |
| Bcin12g06370.1 |  | BGC0000631 | Botrydial\_biosynthetic\_gene\_cluster | 34 | 93.1558935361 | 282 | 1.3e-75 |
| ACD39773.1 | cytochrome\_P450 | BGC0000134 | Radicicol\_biosynthetic\_gene\_cluster | 34 | 87.4524714829 | 259 | 9.2e-69 |
| BAV32150.1 | cytochrome\_P450\_monooxygenase | BGC0001373 | Sordarin\_biosynthetic\_gene\_cluster | 33 | 96.7680608365 | 252 | 8.6e-67 |
| ACZ66254.1 | APS8 | BGC0000304 | Apicidin\_biosynthetic\_gene\_cluster | 28 | 87.072243346 | 182 | 1.1e-45 |
| BAD97696.1 | Aft11-1 | BGC0000003 | AF-toxin\_biosynthetic\_gene\_cluster | 28 | 87.8326996198 | 171 | 2.5e-42 |
| OAQ83757.1 | cytochrome\_P450 | BGC0001358 | Leucinostatins\_biosynthetic\_gene\_cluster | 28 | 85.7414448669 | 168 | 1.6e-41 |
| EAL89317.1 | cytochrome\_P450\_monooxygenase,\_putative | BGC0000686 | Helvolic\_acid\_biosynthetic\_gene\_cluster | 27 | 89.3536121673 | 153 | 5.4e-37 |
| AAZ95016.1 | cytochrome\_P450\_monooxygenase | BGC0000048 | Dothistromin\_biosynthetic\_gene\_cluster | 26 | 88.0228136882 | 143 | 5.6e-34 |
| EAL89312.1 | cytochrome\_P450\_monooxygenase,\_putative | BGC0000686 | Helvolic\_acid\_biosynthetic\_gene\_cluster | 26 | 90.4942965779 | 138 | 1.8e-32 |
| AEE83585.1 | cytochrome\_P450,\_family\_705,\_subfamily\_A,\_polypeptide\_1 | BGC0001313 | Arabidiol\_/\_Baruol\_biosynthetic\_gene\_cluster. | 31 | 39.3536121673 | 93 | 8.7e-19 |
| AEE83588.1 | cytochrome\_P450,\_family\_705,\_subfamily\_A,\_polypeptide\_3 | BGC0001313 | Arabidiol\_/\_Baruol\_biosynthetic\_gene\_cluster. | 27 | 40.4942965779 | 81 | 4.5e-15 |
| ABM21573.1 | CrpE | BGC0000975 | Cryptophycin\_biosynthetic\_gene\_cluster | 25 | 53.2319391635 | 80 | 5.8e-15 |
